# Filtration Characterization Method as Tool to Assess Membrane Bioreactor Sludge Filterability—The Delft Experience

**DOI:** 10.3390/membranes4020227

**Published:** 2014-04-30

**Authors:** Maria Lousada-Ferreira, Pawel Krzeminski, Stefan Geilvoet, Adrien Moreau, Jose A. Gil, Herman Evenblij, Jules B. van Lier, Jaap H. J. M. van der Graaf

**Affiliations:** 1 Department of Water Management, Delft University of Technology, P.O. Box 5048, Delft 2600 GA, The Netherlands; E-Mail: j.b.vanlier@tudelft.nl; 2 Norwegian Institute for Water Research (NIVA), Section Water Supply and Sanitation Technology, Gaustadalléen 21, Oslo N-0349, Norway; E-Mail: pawel.krzeminski@niva.no; 3 Waterboard Hollandse Delta, P.O. Box 4103, Ridderkerk 2980 GC, The Netherlands; E-Mail: s.geilvoet@wshd.nl; 4 Veolia Water Solutions & Technologies, 12 Clematis Ave, Waltham, MA 02453, USA; E-Mail: adrien.moreau@veolia.com; 5 Grundfos BioBooster A/S, Randersvej 22a, Langå DK-8870, Denmark; E-Mail: josean.gil@grundfos.com; 6 Waterboard Groot Salland, P.O. Box 60, Zwolle 8000 AB, The Netherlands; E-Mail: hevenblij@wgs.nl; 7 Witteveen+Bos, van Twickelostraat 2, P.O. Box 233, Deventer 7400 AE, The Netherlands; E-Mail: j.vdgraaf@witteveenbos.nl

**Keywords:** membrane bioreactors, fouling, activated sludge, filterability, delft filtration characterization method (DFCm)

## Abstract

Prevention and removal of fouling is often the most energy intensive process in Membrane Bioreactors (MBRs), responsible for 40% to 50% of the total specific energy consumed in submerged MBRs. In the past decade, methods were developed to quantify and qualify fouling, aiming to support optimization in MBR operation. Therefore, there is a need for an evaluation of the lessons learned and how to proceed. In this article, five different methods for measuring MBR activated sludge filterability and critical flux are described, commented and evaluated. Both parameters characterize the fouling potential in full-scale MBRs. The article focuses on the Delft Filtration Characterization method (DFCm) as a convenient tool to characterize sludge properties, namely on data processing, accuracy, reproducibility, reliability, and applicability, defining the boundaries of the DFCm. Significant progress was made concerning fouling measurements in particular by using straight forward approaches focusing on the applicability of the obtained results. Nevertheless, a fouling measurement method is still to be defined which is capable of being unequivocal, concerning the fouling parameters definitions; practical and simple, in terms of set-up and operation; broad and useful, in terms of obtained results. A step forward would be the standardization of the aforementioned method to assess the sludge filtration quality.

## 1. Introduction

Membrane bioreactor (MBR) technology produces a largely disinfected effluent [1] with reuse potential but the technology consumes quite some energy [1,2]. When analyzing the several specific energy components, membrane aeration is a major energy consumer. Krzeminski *et al*. [2] investigated the energy consumption of two full-scale submerged MBRs, which amounted to 1.05 kW·h·m^−3^ and 0.84 kW·h·m^−3^. In these MBRs membrane aeration was responsible for 57% and 37%, respectively, of the total specific energy. In a full-scale side-stream MBR, consuming a total of 0.97 kW·h·m^−3^, membrane aeration and feed pumps components were responsible for 35% and 43%, respectively [2]. Both in submerged and side-stream MBRs the abovementioned energy components are intended to remove or minimize fouling. Therefore, the major cause of high energy consumption in MBR technology is the prevention and removal of membrane fouling.

Fouling can be defined as the process leading to deterioration of the membrane flux due to surface or internal blockage of the membranes [1]. The established methodology to research membrane fouling is to simulate the filtration process on lab-scale. The well-defined and controllable circumstances that can be created in a lab-scale research are mainly suitable for gaining fundamental knowledge that can subsequently be applied in practice [3]. However, the full-scale MBR fouling process cannot be simulated by this approach, due to the following reasons: the hydraulic circumstances and spatial differences prevailing in full-scale membrane modules distinctly differ from lab-scale modules; lab-scale set-ups generally do not have access to real municipal wastewater and the generally applied synthetic wastewater is not able to reproduce the dynamic quality of the activated sludge present in full-scale installations; MBR fouling consists of short-term and long-term components, the latter is particularly difficult to simulate because it manifests itself on a time-scale of weeks or months and is dependent on the membrane cleaning procedures [4]. Consequently, each MBR installation produces a unique fouling, which cannot be totally reproduced in another MBR installation. Therefore, fouling results from different MBR installations cannot be compared unequivocally with each other.

To overcome the above limitation, a methodology was developed that takes actual full-scale conditions into account. Fouling is a complex process, where three main factors interact: membrane properties, membrane operation, and activated sludge properties [5]. If the membrane properties and membrane operation remain constant, *i.e*., if membrane filtration occurs under well-defined and constant hydraulic conditions, differences in filtration results can be attributed exclusively to the activated sludge properties. Therefore, instead of comparing fouling results, the different MBR installations would be compared through the activated sludge filtration quality, which would be measured following always the same procedure. The latter approach has been applied by various authors leading to the definition of several filterability measurement methods [6,7,8,9,10]. Different parameters, such as the maximum critical flux and filterability, were defined, aiming at quantifying and sometimes qualifying the fouling potential of MBR activated sludge. Each developed method makes use of its own and unique way of fouling assessment. Furthermore, no standard method exists to assess the filtration quality of the activated sludge. Clarification concerning the advantages and disadvantages of several filterability measurement methods, in particular the Delft Filtration Characterization method (DFCm), is therefore required. This article reviews the usefulness of filterability measurements, after a decade of practice, and how to proceed.

## 2. Results and Discussion

### 2.1. Available Methods to Measure Filterability

The most applied methods to measure filterability and other relevant fouling parameters, such as critical flux and resistance, are described in Table 1. The respective installations and operation are described in Table 2. The methods reviewed in Table 1 and Table 2 make use of a filtration test cell and a cross-flow operation mode. The only exception is the Sludge Filtration Index [10] which applies a dead-end filtration mode. Other methods to express the fouling potential of the sludge, based on dead-end filtration and data observation are described by Geilvoet [4], Judd [5], and de la Torre [11].

Le Clech *et al*. [6] proposed to use critical flux as a fouling indicator, which would simultaneously provide a guide value for a suitable operational flux (Table 1). The approach has a valuable practical goal useful for full-scale operation; however, the method itself has two weaker points: the extensive duration of the test, estimated to about 5 h and the definition of critical flux (Table 1). In practice, the critical flux (*J*_c_) does not remain constant; therefore three proposals are made for the *J*_c_ definition, as follows: 1: when d*P*/d*t* < 0.1 mbar·min^−1^; 2: when Δ(d*P*/d*t*)/Δ*J* becomes discontinuous; 3: when, the relation between *J versus P*_ave_ is no longer linear. The duration of the test and the several critical flux definitions, reduce the applicability of the method in full-scale MBR practice.

Evenblij *et al*. [7] developed the DFCm aiming at measuring the filterability of the MBR activated sludge under clearly defined conditions. The DFCm is based on Darcy’s law and comprises a single test lasting about 30 min. The DFCm is a short-term test and measures reversible fouling, *i.e*., the fouling mainly caused by the cake layer filtration mechanism. The definitions used for fouling assessment are similar to those described by Kraume *et al*. [3] listed in Table 3. More details concerning the DFCm are provided in Section 2.2.

**Table 1:** Methods to qualify and quantify the fouling potential in Membrane Bioreactors (MBRs).

Method	Critical flux determination by flux-step method [6]	Delft Filtration Characterization Method (DFCm) [7]	MBR-VITO fouling measurement [8]	Berlin Filtration Method (BFM) [9]	Sludge Filtration Index (SFI) [10]
Fouling Parameter	Critical flux	Filterability	Resistance	Critical flux	Filterability
Principle	Flux (*J*) is increased stepwise until critical flux is obtained	Single TMP filtration measurement at constant supra-critical *J*	Sequence of filtrations steps at constant TMP followed by physical cleaning steps	Flux is increased and subsequently decreased stepwise	Single dead-end filtration through paper filter, relying on gravity filtration
Definitions	Critical flux (*J*_c_): highest flux for which the trans-membrane pressure remains constant	Filterability: fouling potential from the MBR activated sludge. Δ*R*_20_: additional membrane resistance obtained when 20 L·m^−2^ of permeate are produced, following the DFCm. Scale defined between Δ*R*_20_ and sludge filtration quality	Reversible fouling: obtained when operating at an air flow rate of 40 mL·min^−1^; removed by 10 min relaxation and air flow rate of 100 mL·min^−1^.Irreversible fouling: obtained by operating at an air flow rate of 80 mL·min^−1^	Critical flux (*J*_c_): highest flux for which the permeate pressure remains constant.Irreversible fouling: existence of irreversible fouling when hysteresis loop does not present similar values	Filterability: defined as the specific value of the SFI, calculated as the measured time, divided by the MLSS concentration of the sample
Data processing	TMP based parameters in each flux-step: initial TMP increase (Δ*P*_0_); rate of TMP increase d*P*/d*t*; average TMP (*P*_ave_)	Data processed as increased membrane resistance, based on Darcy’s law, see Section 2.2.1.	Data processed as permeability subsequently used to obtain total resistance, further subdivided according to the resistance in series model	Pressure of permeate and applied *J*	Required time to produce specific volume of supernatant; Mixed Liquid Suspended Solids (MLSS) determination
Application	*Ex situ*	*Ex situ* (also possible *in situ*, see Section 2.2.4.1)	*Ex situ* and *In situ*	*In situ*	*Ex situ*
Applicability	Measures removable fouling	Measures removable fouling	Measures removable fouling and attempts to quantify the irremovable fouling	Measures removable fouling and qualifies irremovable fouling	Attempt to quantify filterability-removable fouling
Duration	5 h	30 min	1–2 h	2–3 h	10 min
Usefulness	Guide value for suitable operating flux	Quantify fouling potential	Establishes fouling potential; info concerning need of physical or chemical cleaning	Guide value for suitable operating flux; info concerning irreversible fouling	Information on dewatering properties of the sludge

**Table 2:** Installation/operation of methods to qualify/quantify the fouling potential in MBRs.

Method	Critical flux determination by flux-step method [6]	Delft Filtration Characterization Method (DFCm) [7]	MBR-VITO fouling measurement [8]	Berlin Filtration Method (BFM) [9]	Sludge Filtration Index (SFI) [10]
Installation	40 L bioreactor. Vertical mounted submerged tubular membrane; pore size 0.2 µm. Constant cross-flow of air: bioreactor air-flow 4 L·min^−1^ and module air-flow 6 L·min^−1^	40 L bioreactor. Side-stream membrane; pore size 0.03 µm	Submerged membrane. Presently, several types of tubular membranes are proposed with a pore size from 0.1 to 0.01 µm. Cross-flow of air; fixed values varying according to filtration and physical cleaning steps	Submerged Ultra-filtration flat-sheet membranes with a total filtration surface of 0.025 m^2^ and space between plates of 7 mm; flat-sheet module supplied with aeration	Buchner funnel, with specific paper filter. The sample is mixed through a blade agitator. Volume of produced supernatant is measured and time of production recorded
Method operation	Permeation rate incrementally increased and the pressure change continuously monitored. Step duration: 15 min Step height: 2 L·m^−2^·h^−1^	Sludge filtration at *J* of 80 L·m^−2^·h^−1^ and sludge cross-flow velocity of 1 ms^−1^	(1) Start up: air flow rate of 100 mL·min^−1^; (2) Filtration step to establish membrane resistance and removable fouling: constant TMP of 0.1 bar; air flow rate of 40 mL·min^−1^; (3) Physical cleaning: 10 min relaxation; air flow rate of 100 mL·min^−1^; (4) Filtration steps to establish irremovable fouling: constant TMP of 0.1 bar; air flow rate of 80 mL·min^−1^; (5) At least 10 cycles to establish irremovable fouling with physical cleaning of 3 min relaxation and air flow of 100 mL·min^−1^ in between	Sequence of 5 min filtration steps at constant flux and aeration Specific aeration demand (SAD) of 3.5 m^3^/m^2^·h; Relaxation between filtration steps of 2 min; Flux steps of 3 L·m^−2^·h^−1^ with variable initial step of 5 to 8 L·m^−2^·h^−1^	A 500 mL sludge sample, previously tempered to 20 °C, is placed on the filter and mixed at 40 rpm. The time to produce 100 mL to 150 mL of supernatant is used to calculate the specific value of the SFI. The MLSS concentration of the sample is measured
Cleaning protocol	Backwash with permeate for 5 min at 50–75 mbar. *Ex situ* chemical cleaning with NaOCl (0.5 wt %) at 50 °C for 20 h	Forward flush of water at cross-flow velocity >5 ms^−1^. *In situ* chemical cleaning with Na OCl 500 ppm	Physical cleaning with fixed duration and air flow rate of 100 mL·min^−1^ depending on the operation step. *Ex situ* chemical cleaning, NaOCl at 500 ppm for 2 h	*Ex situ* chemical cleaning with solution of 1% active chlorine	No cleaning protocol

**Table 3:** Types of fouling. Adapted from Kraume *et al*. [3] in Geilvoet [4].

Fouling type	Fouling rate (mbar/min)	Time interval	Cleaning
Reversible fouling	0.1–1	10 min	Mechanical
Irreversible fouling	0.001–0.1	Weeks, months	Chemical
Long-term irreversible fouling	0.0001–0.001	Several years	Impossible

The MBR VITO fouling measurement [8] attempts to quantify reversible and irreversible fouling. The method operation consists of a sequence of filtration steps with several mechanical cleanings (Table 2). Therefore, considering that irreversible fouling, according to Kraume *et al*. [3], takes weeks or even months to occur and that, in order to quantify it, a chemical cleaning step should be included, the assumptions made by the VITO fouling measurement are arguable. Nevertheless, the ability to differentiate between the need for a mechanical and/or a chemical cleaning in full-scale MBRs, is of great interest. Such differentiation is possible, due to the several and differentiated mechanical cleanings steps applied. Where the sequential mechanical cleaning steps are not successful in fouling remediation, chemical cleaning remains the only option.

The Berlin Filtration Method (BFM) [9] overcomes the issue of quantifying irreversible fouling by qualifying it, *i.e*., instead of attempting to quantify a phenomenon that takes weeks or months to develop, the method analyses the activated sludge quality by analyzing the hysteresis properties of the activated sludge. After a sequence of filtration and relaxation steps (Table 2), if the sludge recovers to its initial results, the sludge does not present irreversible fouling and vice-versa. The aforementioned approach is logical and shows the limits of what short-term tests are able to say concerning irreversible fouling. Nevertheless, the BFM also proposes the critical flux as fouling parameter, once more raising the issue of a suitable and practical definition for MBR operation.

The Sludge Filtration Index (SFI) [10] is the most straight forward method mentioned in this article, particularly due to the simplicity of the applied installation, operation and data processing. However, the operation relies on dead-end filtration due to gravity, which is inexistent in full-scale MBRs. Therefore, it is arguable if the method actually provides a quantification of the sludge dewaterability, instead of the sludge filterability as it aims.

The Critical flux determination and the BFM methods propose the critical flux as fouling parameter, while the DFCm, VITO fouling measurement and SFI methods measure fouling through filterability/resistance (Table 1). The critical flux parameter has the advantage of representing the maximum operational flux in MBR operation, which is particularly important when the MBR is applied to produce water for reuse. However, there should be one agreed definition for critical flux, which should produce results confirmed by MBR practice. The parameter of filterability/resistance is an activated sludge quality parameter, providing useful information but not directly applicable to MBR operation.

The information provided by each method varies (Table 1). The Critical flux determination, DFCm, VITO fouling measurement and BFM all measure reversible fouling. Concerning the SFI, the question remains if the method provides a fouling measurement or a sludge dewatering parameter. The VITO fouling measurement establishes the need of a physical or chemical cleaning in MBR operation and aims to quantify irreversible fouling, the latter being as aforementioned arguable. The BFM successfully identifies the existence of irreversible fouling, *i.e.*, qualifying the fouling potential without quantifying it.

The Critical flux determination, DFCm, VITO fouling measurement and SFI are *ex situ* methods (Table 1). The DFCm and VITO fouling measurement can also be applied *in situ*, while the BFM is an *in situ* method (Table 1). The *in situ* methods are more likely to preserve the activated sludge characteristics. However, all methods rely on a specific membrane with a particular operational protocol, which differs from the MBR installation. Therefore *in situ* methods might change the sludge characteristics when measuring the fouling potential, even if in a lower extent than *ex situ* methods. If the method aims to obtain the critical flux, which can be directly applicable to MBR operation, the issue of preserving the original sludge characteristics becomes more significant.

The duration and complexity of the operational protocol are important issues for practical applications. The SFI and DFCm are the fastest and simplest operational methods (Table 1). The Critical flux determination and BFM have less complex operational protocols than the VITO fouling measurement (Table 2).

The methods here described are capable of, as follows: quantifying reversible fouling, identifying the need of physical or chemical cleanings, identifying the existence of irreversible fouling, preserving as much as possible the activated sludge characteristics, being fast and simpler enough to enable its use in MBR full-scale practice. Nonetheless, none of the reviewed method reunites all of the advantages in one single method.

### 2.2. Delft Filtration Characterization Method

**Figure 1:**
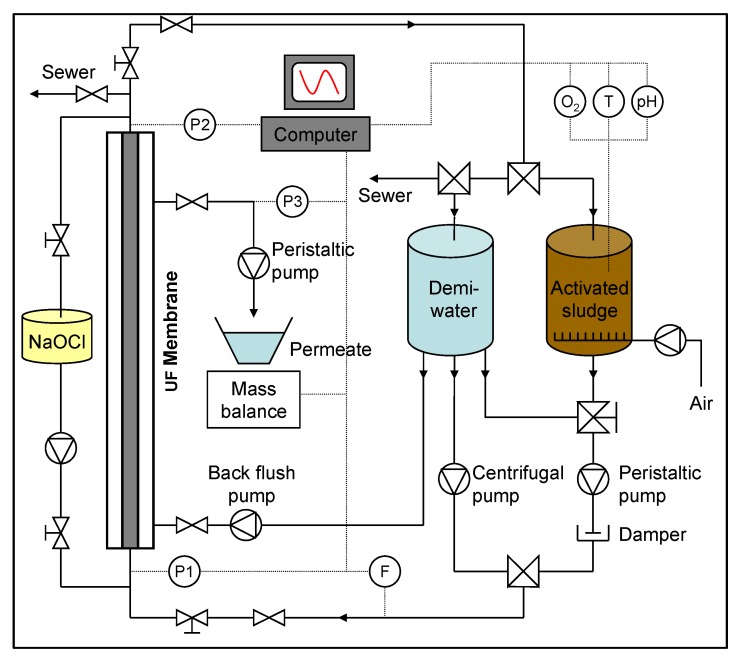
Scheme of the Delft Filtration Characterization Installation (DFCi) [4]. Reprinted with permission from [4]. Copyright 2010 Delft University of Technology.

The Delft Filtration Characterization installation (DFCi), and the measuring protocol, the Delft Filtration Characterization method (DFCm), are described in Evenblij *et al*. [7]. Figure 1 shows the scheme of the DFCi.

#### 2.2.1. Output and Data Processing

The measuring protocol consists of three basic steps, as follows: membrane resistance determination, activated sludge filtration and membrane cleaning. The main step of the measuring protocol is the activated sludge filtration step. The following activated sludge parameters, namely dissolved oxygen (DO) concentration, pH and temperature, and process parameters, namely transmembrane pressure (TMP), flux, and cross-flow velocity, are continuously monitored during the activated sludge filtration step. In the output, these parameters are plotted against the specific produced permeate volume (L·m^−2^). The resulting figures are used to control the development of the activated sludge filtration step online.

**Figure 2:**
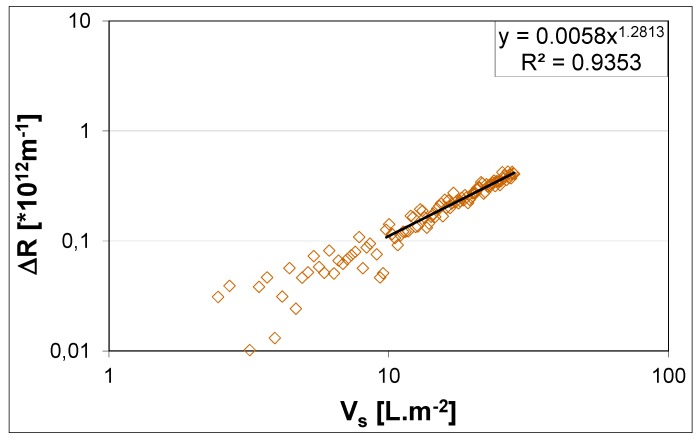
Added resistance according to permeate volume production per membrane area [12]. Reprinted with permission from [12]. Copyright 2011 Delft University of Technology.

The total resistance (*R*_t_) is calculated according to Darcy’s law. The calculation of *R*_t_ is preceded by a flux and permeate viscosity temperature correction. It is assumed that *R*_t_ is the sum of membrane resistance (*R*_m_) plus the resistance imposed by the cake layer built up on the membrane during sludge filtration, referred to as fouling resistance or added resistance (Δ*R*). In the calculation of Δ*R*, *R*_m_ is assumed as the initial value of resistance, *i.e*., the first obtained value of Δ*R* in the activated sludge filtration step. The main output of the DFCi consists of a graph that plots added resistance (Δ*R*) caused by cake layer filtration, as a function of specific permeate production (Vs), as exemplified by Figure 2.

**Figure 3:**
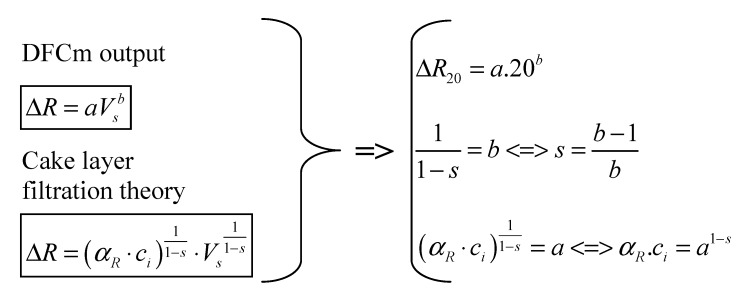
Processing of DFCm output (Adapted from Geilvoet [4] in Lousada-Ferreira [12]).

Notes: Key: Δ*R*: Total cake layer resistance (m^−1^); *a*,*b*: Absolute values obtained in each filtration experiment; *V*_s_: Specific permeate production (L·m^−2^); α_R_: Specific cake resistance (m·kg^−1^); *c*_i_: Concentration of cake layer particles (kg·m^−3^); s: Compressibility coefficient (-); Δ*R*_20_: Total cake layer resistance obtained after 20 L of permeate per m^2^ membrane area are produced (m^−1^).

The calculated values of added resistance are then used to establish a fouling tendency through a power law equation, as explained by Geilvoet [4] (Figure 2). The obtained mathematical expression is then used to calculate the Δ*R*_20_ parameter, α_R_ × c_i_ product and s coefficient. The Δ*R*_20_ is the resistance obtained after extracting 20 L of permeate per membrane area [13], which takes about 15 min. The Δ*R*_20_ parameter, α_R_ × c_i_ product and s coefficient are obtained by fitting the cake layer filtration theory to the DFCm output, as presented by Geilvoet [4] and shown in Figure 3. The Δ*R*_20_ parameter, contrary to the α_R_ × c_i_ product and s coefficient, does not have a direct physical meaning. The parameter was defined to simplify the comparison between filtration curves, assuming that after 15 min of filtration the measurement is stable.

Figure 3 shows how to obtain the Δ*R*_20_ parameter, α_R_ × c_i_ product and s coefficient from single sludge filtration curves. Linear correlation coefficients between Δ*R*_20_ and α_R_ × c_i_ product results are of 0.95 to 0.98 [12,14], indicating that the total cake layer resistance is basically determined by the mass involved and its specific cake resistance. Additionally, in theory, the compressibility coefficient varies between 0 and 1, indicating respectively no compression to total compression. In the DFCm the compressibility results are mainly between 0 and 0.3 [12,14], which shows that hardly compressible cake layers are obtained. Therefore, the DFCm method produces a hardly compressible cake layer, where the cake layer mass and specific cake resistance are the main contributors of the total measured resistance. Furthermore, the Δ*R*_20_ is a fairly good indicator of the method results. 

A classification linking the assessed Δ*R*_20_ and MBR activated sludge filterability was defined by Geilvoet [4], resulting from the weekly monitoring of one full-scale MBR during one year [15] and is shown in Table 4.

**Table 4:** Δ*R*_20_ values and corresponding MBR activated sludge filterability-for standard DFC_m_ measuring protocol [4].

Sludge quality	Δ*R*_20_ (×10 ^12^ m^−1^)	
	Minimum	Maximum
Good	0	0.1
Moderate	0.1	1
Poor	1	–

#### 2.2.2. Installations and Locations

During the 10 years of the DFCm practice three DFCis were built, further designated as DFCi I to III. The DFCi I was permanently stationed at the TU Delft water lab, while the DFCi II and III are mobile. The design is identical for all three installations; however, DFCi II and DFCi III were developed and built with increasing mobility and assembly easiness.

The DFCm was extensively applied in weekly measurements at full-scale MBR installations around Europe [12,14,16], further designated as MBR A to MBR F. The aforementioned Waste Water Treatment Plants (WWTP) have a biological capacity from 7000 to 80,000 population equivalent and a total membrane area from 2436 to 84,480 m^2^. In all visited MBR installations, information concerning characteristics of the MBR such as design, influent quality, activated sludge quality, effluent quality, and MBR performance is requested to the plant operators. In the full-scale weekly campaigns, the DFCi is transported to the MBR installations and is applied as an *ex situ* measurement tool, or exceptionally, as an *in situ* tool.

The DFCm was also applied in lab environment, using the DFCi as a lab-scale research unit. For the latter experiments the activated sludge is collected at MBR full-scale installations and transported to the lab.

#### 2.2.3. Sampling

At the full-scale weekly campaigns, when the DFCm is applied as an *ex situ* measurement tool, the sludge samples are preferably collected in the upper decks of the MBR installations, from central areas of the MBR tanks. In membrane tanks with submerged membranes, the activated sludge is collected from the top of the tank. When exceptionally the DFCm is used *in situ*, the DFCi sludge pump is connected directly to the full-scale membrane tank sludge. During the activated sludge filtration step, the concentrated sludge is returned to the full-scale membrane tank.

When the DFCi is applied as lab-scale research unit, the collection of the activated sludge samples follows the same procedure as applied when the DFCi is applied as *ex situ* tool. Geilvoet [4] studied the consequences of lack of DO to the activated sludge filterability. The author showed that MBR sludge filterability would decrease without aeration, *i.e*., an increase in the Δ*R*_20_ value from 0.05 to 3.3 × 10^12^ m^−1^ was measured, when the activated sludge was kept without DO for 4 days. However, the activated sludge showed a recovery rate 12 times faster than the degradation rate. After a period of 6 h of aeration, the activated sludge presented a Δ*R*_20_ of 0.7 × 10^12^ m^−1^. To overcome the filterability decrease, due to the transport of the samples from the MBR installation to the lab, samples are submitted to aeration, according to the recovery rate obtained by Geilvoet [4] before being submitted to the DFCm measurement.

#### 2.2.4. Evaluation

##### 2.2.4.1. Accuracy

The DFCi was applied as an *in situ* tool at MBR C, in July 2007. The activated sludge and permeate characteristics at MBR C are shown in Table 5. The filterability results obtained in the referred campaign are shown in Table 6.

Table 6 shows that, even considering variations of filterability during one day, the average deviation for Δ*R*_20_ per day is always below 0.1 × 10^12^ m^−1^. Influent daily variations are expected, which is confirmed by the slight variations in pH and temperature of the MBR activated sludge shown in Table 6. The DFCm is capable to follow these fluctuations leading to changes in the order of 0.01–0.02 for Δ*R*_20_, which leads to an accuracy of approximately 10%.

**Table 5:** Activated sludge and permeate characteristics.

Date	4 July 2007	5 July 2007	6 July 2007
Activated sludge	–	–	–
MLSS g/L	14.5	14.6	14.4
Permeability L/m^2^·h·bar	193	199	186
Permeate	–	–	–
COD mg/L	21.7	15.9	18.1
NH_4_-N mg/L	0.01	0.02	0.02
NO_3_-N mg/L	3,3	3,7	4
PO_4_-P mg/L	0.18	0.28	0.36

**Table 6:** Filterability, as Δ*R*_20_, temperature and pH of MBR activated sludge.

Day-Month-Year	Hour:Minute	Δ*R*_20_ (10^12^ m^−1^)	Δ*R*_20_ Standard deviation(daily)	pH	*T* (°C)
4 July 2007	8:27	0.05	0.098	7.1	19.7
4 July 2007	9:31	0.06		7.2	19.7
4 July 2007	10:51	0.08		7.2	19.7
4 July 2007	11:48	0.22		7.1	19.6
4 July 2007	13:09	0.26		7.1	19.5
5 July 2007	8:27	0.21	0.06	6.7	17.8
5 July 2007	10:18	0.08		6.4	17.7
5 July 2007	12:05	0.08		6.1	17.8
5 July 2007	12:59	0.07		6.3	17.8
5 July 2007	13:42	0.16		6.4	17.8
6 July 2007	8:30	0.11	0.025	6.6	18.9
6 July 2007	9:32	0.16		6.5	18.9
6 July 2007	10:21	0.13		6.5	18.9

##### 2.2.4.2. Reproducibility

**Figure 4:**
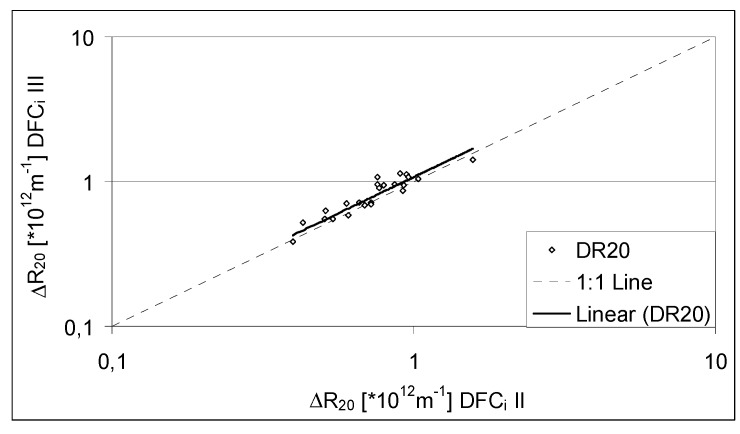
Filterability, as Δ*R*_20_, obtained at two Delft Filtration Characterization Installations: DFCi II and DFCi III. (Adapted from Krzeminski [14]).

A full-scale campaign at MBR D was performed, where activated sludge samples simultaneously collected were submitted to the DFCm in the DFCi II and III [14]. The obtained results are shown in Figure 4.

Figure 4 shows that there is a strong correlation between results obtained in the two DFCis. The Pearson coefficient, between the two sets of results, is of 0.907. The results provided by the DFCi III were in average 7% higher than the results obtained in the DFCi II. As deduced in Section 2.2.4.1, the accuracy of the DFCm is of about 10% therefore the DFCm results are considered reproducible, irrespective of the used installation.

##### 2.2.4.3. Reliability

The average and standard deviation of the filterability as well as the MBR operation stability, obtained at several full-scale MBRs, are shown in Table 7. The MBR operation was defined as “steady” when the effluent quality parameters were below the discharge limits and if the permeability remained stable, during the weekly campaign [16].

**Table 7:** Filterability, as Δ*R*_20_, and MBR installation stability (Adapted from Moreau [16] and Krzeminski [14]).

MBR installation	Month Year	Δ*R*_20_ (10^12^ m^−1^)		MBR operation stability
		Average	Standard deviation	
A	February 2007	0.97	0.11	Steady
	April 2008	3.01	1.47	Unsteady
	August 2008	0.31	0.07	Steady
B	March 2007	0.56	0.04	Steady
	September 2008	0.08	0.02	Steady
C	July 2007	0.12	0.07	Steady
	November 2008	0.43	0.07	Steady
D	February 2007	0.31	0.12	Steady
	June 2008	0.05	0.05	Steady
	January 2009	0.30	0.12	Steady
	July 2009	0.14	0.07	Steady
	February 2010	0.77	0.14	Steady
E	June 2008	0.18	0.04	Steady
	February 2009	2.72	0.41	Unsteady
	August 2009	0.04	0.01	Steady
	March 2010	0.95	0.13	Steady
F	June 2008	0.17	0.04	Steady
	February 2009	3.46	0.37	Unsteady
	August 2009	0.04	0.00	Steady
	March 2010	0.75	0.11	Steady

Table 7 shows that stable reactor operation corresponds to Δ*R*_20_ values below 1 × 10^12^ m^−1^ with a maximum standard deviation of 0.14 × 10^12^ m^−1^. On the opposite, when the operation is unstable the obtained Δ*R*_20_ values are above 1 × 10^12^ m^−1^ and present a standard deviation between 0.3 × 10^12^ and 1.5 × 10^12^ m^−1^. The aforementioned results show that the filterability measurements are consistent with the operation state of the MBR and therefore provide reliable information. In fact, filterability is the connecting parameter between membrane bioreactor ‘biology’ and membrane operation.

##### 2.2.4.4. Applicability

The DFCm measures the filterability of an activated sludge sample, which is one of the starting points for a satisfactory MBR filtration process [4]. If filterability, with its dynamic changes, is properly evaluated, the process operation can be optimized. Furthermore, the DFCm can be useful to research how filterability can be influenced by, as follows: MBR configuration [17]; MBR design, in particular hydraulic retention time [18] and recirculation [19]; membrane configurations [18,20]; wastewater influent characteristics [20,21,22]; activated sludge characteristics, such as temperature [18,21], mixed liquid suspended solids [18,23], viscosity [24], floc size [19], soluble organic fractions [18], sludge morphology and relative hydrophobicity [25]; operational parameters, such as sludge retention time [18,26], food to mass ratio [18], substrate addition [27], and dissolved oxygen concentrations [28].

The DFCm is a short-term filtration experiment. When the sludge filtration step is initiated the membrane is still clean and the initial fouling mechanism will be pore blocking, which will shift to cake layer filtration depending on the amount of accumulated substances. Jiang *et al*. [29] performed filtration tests in a set-up with a side-stream membrane, fluxes of 52 to 72 L·m^−2^·h^−1^, and reported that the main fouling mechanism changed from pore blocking to cake filtration after 8 s. Considering the high MLSS concentration in MBR sludge, usually superior to 6 g/L [12], and the high flux applied in the DFCm, of 80 L·m^−2^·h^−1^, it is expected that the dominant fouling mechanism is cake filtration. Furthermore, in the DFCm the resistance is quantified based on Darcy’s law, therefore cake filtration is only mechanism taken into account for the calculations. As a short-term experiment, the DFCm will mainly measure reversible fouling.

The long-term performance of an MBR installation will be mainly determined by the irreversible and irrecoverable fouling. The irreversible fouling is expected to be a consequence of the removal efficiency of the reversible fouling [4]. A relation between filterability and irreversible fouling can be empirically analyzed through the developments of filterability and permeability, of the considered MBR plant. In the aforementioned case, the DFCm allows the evaluation of the activated sludge properties in the filtration process and consequently the eventual optimization of the operation conditions, such as filtration and relaxation/backwash protocols. Another optimization possibility deriving from frequent filterability measurements at an MBR installation is to allow operators to take advantages of good filterability periods to improve the energy efficiency of the plant, by for instance prolonging the filtration protocol. Additionally, frequent filterability measurements could also act as an early warning system for operators and as a membrane aeration energy optimization tool.

## 3. Conclusions

The available methods to measure fouling are, at present, fast enough to become practical, capable of satisfactory quantify removable fouling and identify the existence of irremovable fouling, capable of producing results, which can eventually lead to the optimization of full-scale MBR operation. Nevertheless, each of the described methods, presents one or more of the following disadvantages: uncertainty concerning the definition of the selected fouling parameter, such as the critical flux parameter; limitation regarding the type of fouling, reversible and irreversible, that can actually be quantified through short-term measurements; complex installations and operational steps; incapability to reproduce the cross-flow membrane filtration operation. 

Concerning the DFCm, the accuracy of the method lies in the error range of 10%, leading to a positive evaluation of the reproducibility and reliability of the DFCm results. Results obtained in six MBR full-scale plants, and in three DFCm installations, showed that results are reproducible and reliable. The DFCm is a short-term measurement, measuring reversible fouling, which can provide useful information for MBR operation optimization.

Nevertheless, a fouling measurement method is still to be defined which is capable of being unequivocal, concerning the definitions of its fouling parameters; practical and simple, in terms of set-up and operation; broad and useful, in terms of obtained results. A step further would be the standardization of the aforementioned method capable of assessing the filtration quality of the sludge.
